# Two-year outcomes following concomitant ACL and extra-articular ligament reconstruction: a nationwide analysis of over 5,000 patients

**DOI:** 10.1007/s00402-026-06422-0

**Published:** 2026-08-10

**Authors:** Rayyan Abid, Robert J. Burkhart, Andrew J. Moyal, Jeremy M. Adelstein, Mark Kodsy, Jacob G. Calcei, James E. Voos, John M. Apostolakos

**Affiliations:** University Hospitals Cleveland Medical Center, Cleveland, USA

**Keywords:** ACL reconstruction, Extra-articular ligaments, Postoperative outcomes, Emergency department utilization, Large database study

## Abstract

**Purpose:**

The anterior cruciate ligament (ACL) is the most commonly injured and reconstruction ligament in the United States. Associated injuries are common, oftentimes including components of the extra-articular knee. However, complications following concomitant ACL and extra-articular ligaments (EAL) has not been defined in a large-scale, population-based sample. We aimed to analyze outcomes following concomitant ACL and EAL reconstruction using the TriNetX nationwide database.ep

**Methods:**

Deidentified data from the TriNetX Collaborative Network was used to identify all patients who received ACL and EAL reconstruction on the same day. Demographic information including age, sex, race, and geographic data were collected. Rates of medical and orthopaedic complications were analyzed at 30, 90, and 180 days and at 2 years. A propensity matched sub-analysis comparing outcomes patients with concomitant and staged repair was performed. Patients were identified using Current Procedural Terminology (CPT) coding.

**Results:**

We identified 5944 patients who received concomitant ACL and EAL reconstruction with postoperative outcomes available at 30, 90, and 180 days. 5040 patients had data available at 2 years. Serious medical complications like surgical site infection, deep vein thrombosis, acute kidney failure, and sepsis were rare at each point of follow-up. Rates of revision surgery were low at short-term follow-up but were prevalent for both procedures at 2 years (> 2%). The most common orthopaedic complications osteoarthritis, implant/graft failure, and knee instability. However, these incidences remained below 7.5% at each interval. Outcomes were similar for staged and concomitant reconstruction, but osteoarthritis, implant/graft failure, and knee instability were significantly more common in the former group at various points.

**Conclusions:**

Concomitant ACL and EAL reconstruction demonstrated negligible rates of serious medical complications. While rates of osteoarthritis, instability, and graft failure increased at each point, these were generally comparable to independent ACL and EAL procedures. While there was little difference in outcomes between staged and concomitant reconstruction, the latter should be preferred due to lower rates of osteoarthritis, graft failure, and instability.

**Level of evidence:**

Level III.

## Introduction

The anterior cruciate ligament (ACL) is the most commonly injured and reconstruction ligament in the United States 1. Left untreated, these injuries often lead to a number of complications, including instability of the knee, meniscal and articular cartilage damage, and eventual osteoarthritis 2. Thus, active patients are typically treated with arthroscopic reconstruction and face few complications at short and long-term follow-up [3]. ACL are tears are commonly associated with concomitant injuries, typically to the articular cartilage. However, extra-articular injuries are also often found along with ACL tears. For example, 10% of complex knee injuries involve the ACL and posterolateral corner (PLC) and collateral ligament repair accompanies 12.3% of ACL reconstructions 4,5. The extra-articular knee is critical for its role in supporting surrounding ligaments and overall stability 6,7. Specifically, these structures are responsible for restraining rotational forces, and injury may lead to ACL reconstruction failure if untreated 8–11.

Reporting on outcomes and complications following concomitant repair of ACL and extra-articular injuries within a single cohort is currently limited to case-series and studies with small sample sizes. To our knowledge, there are no studies evaluating these metrics in an individual, large, population-based sample. Thus, the aim of this study is to determine rates of medical and orthopaedic complications following combined ALC and extra-articular ligament (EAL) reconstruction in a nationwide, multicenter sample at 30-day, 90-day, 180-day, and 2-year follow-up. Secondarily, we aim to compare these outcomes among patients with combined versus staged reconstruction at each follow-up interval. We hypothesized that patients undergoing concomitant ACL and EAL reconstruction will have lower complications compared to staged reconstruction at all time points.

## Methods

### Study design

We utilized the TriNetX U.S. Collaborative Research Platform to conduct a multicenter retrospective analysis of outcomes following concomitant ACL and EAL reconstruction. The TriNetX platform has access to health information from 104 healthcare organizations and over 133 million patients across the United States. Due to deidentification and minimum thresholds necessary for patient analysis (10 patients), this data is compliant with § 164.514(b)(1) of the HIPAA Privacy Rule and is exempt from Western Institutional Review Board regulations. All data was found using insurance billing codes including Current Procedural Terminology (CPT) codes and e International Classification of Diseases, 10th Revision (ICD-10) codes.

### Patient selection

**Fig. 1:**
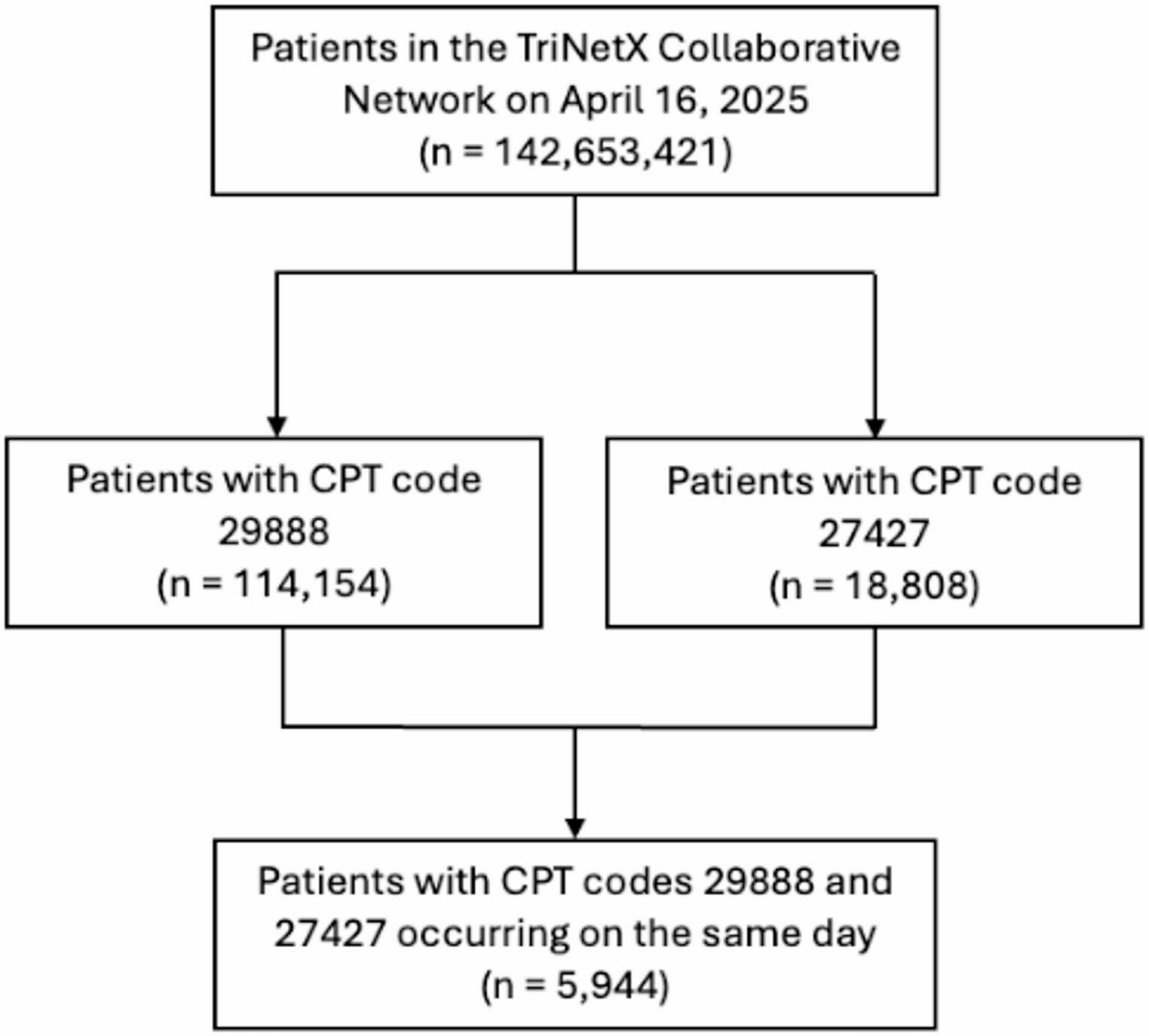
STROBE diagram depicting the patient selection process

Patients in the TriNetX database who underwent ACL and EAL reconstruction were identified using CPT codes 29,888 and 27,427, respectively, as described in a previous database study comparing reconstruction and repair for EAL injuries 12. The cohort was structured to ensure instances of both codes occurred on the same day. We then identified patient demographics, including age, sex, race, and ethnicity. To perform a sub-analysis comparing those with concomitant versus staged reconstruction, we created a second cohort with patients using the same codes described previously but with instances at least one week apart (Fig. 1). These cohorts were then 1:1 propensity matched by age, sex, race, and ethnicity.

### Outcome measures

We evaluated both short-term (30, 90, and 180 days) and long-term (2 years) outcomes for a number of medical and orthopaedic complications. Medical complications included incidence of surgical site infection (SSI), deep vein thrombosis (DVT), sepsis, myocardial infarction, hematoma, and postoperative anemia. Orthopaedic complications included ACL reconstruction following initial treatment, EAL reconstruction following initial treatment, meniscus derangement, ankylosis, lysis of adhesions, partial knee arthroplasty, peroneal nerve injury, osteoarthritis, orthopaedic implant/graft complications, and knee instability. Emergency department utilization following treatment was also evaluated.

### Statistical analysis

All statistical analysis was conducted using tools built into the TriNetX platform. Continuous variables were evaluated using mean and standard deviations and then calculated via Mann-Whitney U test. After propensity matching cohorts, they were compared using odds ratios (OR). For outcomes with frequencies less than or equal to 10 patients, the TriNetX platform automatically rounds up to 10 to protect patient privacy. Outcomes with *p* < 0.05 were considered significant. Bonferroni correction was applied to the sub-analysis comparing staged and concomitant reconstruction, and a *p* < 0.0007 was considered significant.

## Results

### Medical complications

Incidence of significant medical complications were low across both short and long-term follow-up for patients with concomitant reconstruction. Namely, rates of myocardial infarction, pulmonary embolism, postoperative anemia, postoperative hematoma, and acute kidney failure remained below 2% at each interval (Table 1). Emergency department visits increased steadily between 30, 90, and 180-day follow-up. SSI and DVT were rare at 30 days (0.6% and 0.7%, respectively) but demonstrated an appreciable increase at 90-day follow-up (1.3% and 1.0%, respectively).

**Table 1:** Comparative analysis of postoperative complications of 5,918 patients at 30, 90, and 180 days

Outcomes	30 Day *N* (%)	90 Day *N* (%)	180 Day *N* (%)
Surgical Site Infection	33 (0.6%)	70 (1.2%)	79 (1.3%)
Deep Vein Thrombosis	40 (0.7%)	57 (1.0%)	59 (1.0%)
Sepsis	≤ 10 (0.2%)	≤ 10 (0.2%)	≤ 10 (0.2%)
Emergency department visit	163 (2.8%)	215 (3.6%)	287 (4.9%)
Myocardial infarction	≤ 10 (0.2%)	≤ 10 (0.2%)	≤ 10 (0.2%)
Postoperative Hematoma*	≤ 10 (0.2%)	≤ 10 (0.2%)	12 (0.2%)
Postoperative anemia*	13 (0.2%)	16 (0.3%)	19 (0.3%)
Acute Kidney Failure	≤ 10 (0.2%)	11 (0.2%)	15 (0.3%)
Stroke	≤ 10 (0.2%)	≤ 10 (0.2%)	≤ 10 (0.2%)
Pulmonary Embolism	16 (0.3%)	21 (0.4%)	24 (0.4%)
Revision ACL Reconstruction	14 (0.2%)	35 (0.6%)	62 (1.0%)
Revision PLC Reconstruction	18 (0.3%)	24 (0.4%)	43 (0.7%)
Meniscus Derangement	58 (1.0%)	70 (1.2%)	83 (1.4%)
Ankylosis	18 (0.3%)	140 (2.4%)	224 (3.8%)
Lysis of Adhesions	0 (0.0%)	41 (0.7%)	106 (1.8%)
Partial Knee Arthroplasty	0 (0.0%)	0 (0.0%)	0 (0.0%)
Peroneal Nerve Injury	51 (0.9%)	67 (1.1%)	83 (1.4%)
Osteoarthritis	65 (1.1%)	100 (1.7%)	140 (2.4%)
Implant/Graft Complications	180 (3.0%)	231 (3.9%)	291 (4.9%)
Knee Instability	172 (2.9%)	224 (3.8%)	281 (4.7%)

### Orthopaedic complications

Patients with concomitant reconstruction demonstrated low rates of subsequent ACL and EAL procedures at short-term follow-up, remaining below 1% at each interval (Table 2). However, over 3% of patients underwent ACL repair and 2% underwent EAL repair at 2-year follow-up. There were no conversions to partial knee arthroplasty at any point. Meniscus derangement, osteoarthritis, and peroneal nerve injury were also rare but increased steadily between 30, 90, and 180-day follow-up with incidence of osteoarthritis peaking above 5% at 2 years. Rates of ankylosis and lysis of adhesions increased considerably between 30 and 180 days and steadily increased until 2 years. Implant/graft complications and knee instability were the most common outcomes at each point, both nearing a rate of 5% at 2-year follow-up.

**Table 2:** Comparative analysis of postoperative orthopaedic complications at 2 years (*n* = 5,040)

Orthopaedic Outcomes	2 Year *N* (%)
Surgical Site Infection	75 (1.5%)
Deep Vein Thrombosis	55 (1.1%)
Sepsis	≤ 10 (0.2%)
Myocardial infarction	≤ 10 (0.2%)
Postoperative Hematoma*	11 (0.2%)
Postoperative anemia*	28 (0.6%)
Acute Kidney Failure	21 (0.4%)
Stroke	≤ 10 (0.2%)
Pulmonary Embolism	18 (0.4%)
Revision ACL Reconstruction	160 (3.2%)
Revision PLC Reconstruction	99 (2.0%)
Meniscus Derangement	115 (2.3%)
Ankylosis	221 (4.4%)
Lysis of Adhesions	128 (2.5%)
Partial Knee Arthroplasty	0 (0.0%)
Peroneal Nerve Injury	95 (1.9%)
Osteoarthritis	289 (5.7%)
Implant/Graft Complications	375 (7.4%)
Knee Instability	345 (6.8%)

### Concomitant versus staged reconstruction

Incidence of revision surgeries, meniscus derangement, ankylosis, and lysis of adhesions, total knee arthroplasty, and peroneal nerve injury also did not differ significantly at any point (Table 3). There were no significant differences in rates of medical complications between concomitant and staged repair at any follow-up interval (Table 4). Patients with staged repair demonstrated greater rates of knee instability and implant/graft complications at short-term follow-up and osteoarthritis at long-term follow-up, but these differences were also insignificant.

**Table 3:** Orthopaedic Complications following Staged versus Concomitant ACL + EAL

Complications	Concomitant ACL + EAL (*N* = 1,062)	Staged ACL + EAL (*N* = 1,062)	Odds-Ratio (Confidence Interval)	Risk Difference *p* value
Revision ACL				
*30d*	≤ 10 (0.9%)	0 (0.0%)	-	-
*90d*	≤ 10 (0.9%)	≤ 10 (0.9%)	1 (0.4,2.4)	1
*180d*	14 (1.3%)	≤ 10 (0.9%)	1.4 (0.6, 3.2)	0.41
*2 yr*	37 (3.5%)	33 (3.1%)	1.1 (0.7, 1.8)	0.63
Revision PLC				
*30d*	≤ 10 (0.9%)	0 (0.0%)	-	-
*90d*	≤ 10 (0.9%)	≤ 10 (0.9%)	1 (0.4,2.4)	1
*180d*	18 (1.7%)	≤ 10 (0.9%)	1.8 (0.8, 3.9)	0.13
*2 yr*	34 (3.2%)	25 (2.4%)	1.4 (0.8, 2.3)	0.23
Meniscus Derangement				
*30d*	≤ 10 (0.9%)	14 (1.3%)	0.7 (0.3, 1.6)	0.41
*90d*	≤ 10 (0.9%)	16 (1.5%)	0.6 (0.3, 1.4)	0.24
*180d*	15 (1.4%)	17 (1.6%)	0.9 (0.4, 1.8)	0.72
*2 yr*	25 (2.4%)	28 (2.6%)	0.9 (0.5, 1.5)	0.68
Ankylosis				
*30d*	≤ 10 (0.9%)	≤ 10 (0.9%)	1 (0.4,2.4)	1
*90d*	26 (2.4%)	17 (1.6%)	1.5 (0.8, 2.9)	0.17
*180d*	27 (2.6%)	20 (1.9%)	1.4 (0.8, 2.4)	0.30
*2 yr*	44 (4.2%)	32 (3.0%)	1.4 (0.9, 2.2)	0.16
Lysis of Adhesions				
*30d*	0 (0.0%)	≤ 10 (0.9%)	-	-
*90d*	≤ 10 (0.9%)	≤ 10 (0.9%)	1 (0.4,2.4)	1
*180d*	20 (1.9%)	≤ 10 (0.9%)	2.0 (0.9, 4.3)	0.07
*2 yr*	24 (2.3%)	17 (1.6%)	1.4 (0.8, 2.7)	0.27
Total Knee Arthroplasty				
*30d*	0 (0.0%)	≤ 10 (0.9%)	-	-
*90d*	0 (0.0%)	≤ 10 (0.9%)	-	-
*180d*	0 (0.0%)	≤ 10 (0.9%)	-	-
*2 yr*	≤ 10 (0.9%)	≤ 10 (0.9%)	1 (0.4,2.4)	1
Osteoarthritis				
*30d*	14 (1.3%)	19 (1.8%)	0.7 (0.4, 1.5)	0.38
*90d*	21 (2.0%)	23 (2.2%)	0.9 (0.5, 1.7)	0.76
*180d*	28 (2.7%)	35 (3.3%)	0.8 (0.4, 1.3)	0.37
*2 yr*	50 (4.7%)	73 (6.9%)	0.7, (0.5, 1.0)	0.03
Implant/Graft Complications				
*30d*	48 (4.5%)	80 (7.5%)	0.6 (0.4, 0.8)	< 0.01
*90d*	64 (6.0%)	99 (9.3%)	0.6 (0.5, 0.9)	< 0.01
*180d*	79 (7.5%)	112 (10.6%)	0.7 (0.5, 0.9)	0.01
*2 yr*	124 (11.7%)	148 (14.0%)	0.8 (0.7, 1.0)	0.12
Knee Instability				
*30d*	34 (3.2%)	53 (5.0%)	0.6 (0.4, 1.0)	0.04
*90d*	47 (4.4%)	62 (5.8%)	0.7 (0.5, 1.1)	0.14
*180d*	53 (5.0%)	71 (6.7%)	0.7 (0.5, 1.1)	0.10
*2 yr*	76 (7.2%)	91 (8.6%)	0.8 (0.6, 1.1)	0.23
Peroneal Nerve Injury				
*30d*	≤ 10 (0.9%)	≤ 10 (0.9%)	1 (0.4,2.4)	1
*90d*	12 (1.1%)	≤ 10 (0.9%)	1.2 (0.5, 2.8)	0.67
*180d*	14 (1.3%)	≤ 10 (0.9%)	1.4 (0.6, 3.2)	0.41
*2 yr*	14 (1.3%)	11 (1.0%)	1.2 (0.5, 2.7)	0.41

**Table 4:** Medical complications following staged versus concomitant ACL + EAL

Complications	Concomitant ACL + EAL (*N* = 1,062)	Staged ACL + EAL (*N* = 1,062)	Odds-Ratio (Confidence Interval)	Risk Difference *p* value
Surgical site infection				
*30d*	≤ 10 (0.9%)	≤ 10 (0.9%)	1 (0.4,2.4)	1
*90d*	11 (1.0%)	13 (1.2%)	0.8 (0.4, 1.9)	0.68
*180d*	15 (1.4%)	13 (1.2%)	1.2 (0.6, 2.7)	0.56
Deep vein thrombosis				
*30d*	≤ 10 (0.9%)	≤ 10 (0.9%)	1 (0.4,2.4)	1
*90d*	≤ 10 (0.9%)	≤ 10 (0.9%)	1 (0.4,2.4)	1
*180d*	11 (1.0%)	≤ 10 (0.9%)	1.1 (0.4, 2.6)	0.83
Postoperative Hematoma				
*30d*	0 (0.0%)	≤ 10 (0.9%)	-	-
*90d*	≤ 10 (0.9%)	≤ 10 (0.9%)	1 (0.4,2.4)	1
*180d*	≤ 10 (0.9%)	≤ 10 (0.9%)	1 (0.4,2.4)	1
Myocardial infarction				
*30d*	0 (0.0%)	0 (0.0%)	-	-
*90d*	0 (0.0%)	0 (0.0%)	-	-
*180d*	≤ 10 (0.9%)	≤ 10 (0.9%)	1 (0.4,2.4)	1
Postoperative anemia				
*30d*	≤ 10 (0.9%)	≤ 10 (0.9%)	1 (0.4,2.4)	1
*90d*	≤ 10 (0.9%)	≤ 10 (0.9%)	1 (0.4,2.4)	1
*180d*	≤ 10 (0.9%)	≤ 10 (0.9%)	1 (0.4,2.4)	1
Acute kidney failure				
*30d*	≤ 10 (0.9%)	≤ 10 (0.9%)	1 (0.4,2.4)	1
*90d*	≤ 10 (0.9%)	≤ 10 (0.9%)	1 (0.4,2.4)	1
*180d*	≤ 10 (0.9%)	≤ 10 (0.9%)	1 (0.4,2.4)	1
Stroke				
*30d*	0 (0.0%)	0 (0.0%)	-	-
*90d*	0 (0.0%)	0 (0.0%)	-	-
*180d*	0 (0.0%)	0 (0.0%)	-	-
Pulmonary Embolism				
*30d*	≤ 10 (0.9%)	≤ 10 (0.9%)	1 (0.4,2.4)	1
*90d*	≤ 10 (0.9%)	≤ 10 (0.9%)	1 (0.4,2.4)	1
*180d*	≤ 10 (0.9%)	≤ 10 (0.9%)	1 (0.4,2.4)	1
Sepsis				
*30d*	0 (0.0%)	0 (0.0%)	-	-
*90d*	0 (0.0%)	0 (0.0%)	-	-
*180d*	≤ 10 (0.9%)	0 (0.0%)	-	-

## Discussion

Despite the importance of EAL structures in mitigating rates of morbidity following ACL reconstruction, little population-based data exists regarding outcomes following concomitant reconstruction of ALC and EAL injuries. Our analysis generally found low rates of significant medical complications. Certain orthopaedic complications, including knee instability, postoperative implant/graft complications, and osteoarthritis, demonstrated considerable rates of occurrence, increasing steadily at each follow-up interval. Outcomes following staged and concomitant reconstruction are similar, but concomitant reconstruction offers lower rates of implant/graft complications and knee instability at short-term follow-up and osteoarthritis at long-term follow-up.

### Medical complications

Rates of serious medical complications, including SSI, DVT, and pulmonary embolism remained low at both short and long-term follow-up as both incidences never exceeded 1.5% at any point. These results generally align with existing studies analyzing outcomes of EAL and ACL reconstruction. For example, a systematic review of 60 studies and 1747 cases of PLC repair or reconstruction found an overall infection rate of 1.3% in at approximately 3-year follow-up [13]. Varelas et al. found infections in 5 of 275 patients (1.8%) following medial collateral ligament (MCL) reconstruction at minimum 2-year follow-up [14]. Rates of infection following ACL repair has also been reported to range between 0.5 and 1.5% [15–17]. Interestingly, Maheshwer et al. identified only one case of either DVT or pulmonary embolism following isolated PLC reconstruction, an incidence of 0.06%, while our sample exhibited a minimum incidence of 0.7% and 0.3% for DVT and pulmonary embolism at each time interval, respectively 13. However, these rates are lower than the 2% incidence described by Born et al. in a study of 134 multiligamentous reconstructions with thromboprophylaxis 18. Similarly, both rates of complications are generally comparable with past studies describing DVT and pulmonary embolism following ACL reconstruction 19–21.

Incidence of sepsis, myocardial infarction, and stroke were low at each time interval, remaining below 0.6% at each time interval. While rates of these outcomes are poorly described following EAL repair and reconstruction, these figures are similar to those found after ACL surgery. In a consecutive case series of 1,283 patients who underwent ACL reconstruction, Sajovic et al. found only 3 instances (0.23%) of septic arthritis at mean 33 month follow-up [22]. Abram et al. observed 4 cases of myocardial infarction, 2 cases of stroke, and 13 cases of acute kidney injury at 90-day follow-up in a study of 104,255 ACL reconstructions.^23^ Schuster et al. observed only 2 cases of septic arthritis following 458 multiligamentous reconstructions at minimum 3-month follow-up. Postoperative hematoma, postoperative anemia, and acute kidney failure were also very rare throughout the follow-up period, never exceeding 0.6% incidence at any point.

Emergency department (ED) utilization increased at each follow-up interval, with short-term follow-up peaking at 4.9% at 180 days. Again, this outcome is poorly described following EAL reconstruction. However, Kammien et al. found that 8.3% of 81,179 patients with ACL reconstruction had ED visits at 90-day follow-up, nearly double the incidence within our sample. Similarly, Liu et al. found 3.9% of patients utilized healthcare resources at 30-day follow-up in a sample of 26,873 subjects 24. These increased rates in isolated ACL surgery may be due to greater postoperative planning after multiligamentous repair or greater likelihood of inpatient admission following complex repairs in our sample.

### Orthopaedic complications

Rates of revision surgery were low at short-term follow-up, but 3.2% of patients underwent ACL reconstruction and 2% underwent EAL reconstruction at 2-year follow-up. These figures were generally lower than rates of graft failure in past studies of concomitant ACL repair with specific EAL structures. For example, Helito et al. found that 4.6% of 88 patients experienced graft failure following ACL reconstruction with additional anterolateral ligament reconstruction or iliotibial band tenodesis 25. Our results are also comparable to studies of independent ACL surgery. For example, Liuokken et al. found a revision rate of 3.14% in a systematic review of 52,878 ACL reconstruction patients with median 2.3 year follow-up [26].

To our knowledge, no previous studies have defined rates of ankylosis or lysis of adhesions following concomitant ACL and EAL repair. Notably, we found that ankylosis was the second most common complication at 180-day follow-up and remained prevalent at 2 years (4.4%). Similarly, Hettrich et al. had 5.4% rate of arthrofibrosis in 980 patients with ACL reconstruction at 6-year follow-up [27]. Lysis of adhesions was less common than ankylosis at each point of follow-up but was still observed in 2.5% of patients at 2 years. Huleatt et al. demonstrated a 4.5% of patients who received 2424 ACL reconstructions underwent either manipulation under anesthesia or lysis of adhesions. In a systematic review of 199 MCL and posterior oblique ligament reconstructions, D’Ambrosi et al. found arthrofibrosis in only 2 patients (1%). This significant disparity between reported figures may suggest that patients in our sample may be requiring lysis due to the ACL portion of their surgeries and would have required this subsequent procedure regardless of concomitant EAL reconstruction. However, Patel et al. had 16.7% of 134 patients with operative treatment of multiligamentous knee injuries undergo manipulation under anesthesia or lysis of adhesions due to arthrofibrosis 28.

Osteoarthritis and knee instability were prevalent at both short and long-term follow-up, peaking at 5.7% and 6.8% at 2 years, respectively. Both figures were generally higher than previously reported figures following reconstruction of some EAL structures. Stannard et al. found osteoarthritis in 1 patient (2%) at minimum 24-month follow-up for PLC repair 29. However, Jacobs et al. observed that 299 of 3325 (9%) patients with multiligamentous reconstruction were diagnosed with osteoarthritis within 5 years of surgery. Moreover, our data generally agrees with larger samples of patients following ACL reconstruction. For example, Bodkin et al. found that 6.2% of 10,565 patients experienced osteoarthritis at 2 years 30. Similarly, Ferretti et al. determined that adding extra-articular reconstruction to ACL reconstruction does not significantly increase the risk of osteoarthritis at 25 years in a sample of 75 patients.^31^ Implant/graft complications were the most common complications at each time interval. However, incidence varies significantly in past studies of combined ACL reconstructions 13,32–34. Similarly, rates of ACL graft failure in large cohorts are lower than our own 35–37. This disparity may be due to increased strain on ACL grafts due to simultaneous reconstruction and subsequent weakness of EAL structures, leading to greater rates of failure than in independent reconstruction 8,9. Peroneal nerve injury also increased throughout patients’ follow-ups, but this was likely a function of later coding or diagnosis than a true increase in incidence.

### Concomitant versus staged reconstruction

There were no significant differences in rates of orthopaedic or serious medical complications between concomitant and staged ACL and EAL reconstruction. However, staged reconstruction demonstrated considerably greater rates of implant/graft failure at each point of short-term follow-up. This observation aligns with previous studies that have established that residual instability and laxity found in a staged approach increases the likelihood of graft failure in surrounding reconstructed ligaments, especially concerning the PLC 38–41. While others have advocated for a staged approach due to the length of both reconstructions, our results indicate that a potential risk for graft failure can be avoided with a concomitant approach within 180 days 7,29,42. While insignificantly different, patients also experienced nearly double the incidence of knee instability at 30-day follow-up following staged reconstruction, potentially reflecting the laxity found between procedures described in the literature.

Staged reconstruction also had a notably greater risk of osteoarthritis at 2-year follow-up. To our knowledge, there have been no previous reports comparing long-term outcomes between staged and concomitant procedures. This disparity may be due a greater period of uncorrected instability in the staged group. However, it is more likely that patients with staged reconstruction are those who underwent more severe forms of trauma, prompting a staged approach. A greater degree of initial articular damage may be responsible for the greater rates of osteoarthritis within this group. Regardless, our results indicate that there are no significant differences in complications following concomitant and combined ACL and EAL reconstruction.

### Limitations

This study has a number of limitations. TriNetX draws on data from multiple healthcare systems throughout the United States, potentially leading to inconsistences in reporting. Similarly, selection bias may emerge from only considering patients treated within participating hospitals. Reliance on ICD-10 and CPT codes likely obscures unique patient characteristics and other detailed clinical information. Additionally, do to coding limitations, we were only able to identify EAL injuries using CPT code 27,247. This code does not differentiate between specific ligament repairs such as MCL, LCL, or PLC. As a result, we were unable to comment on the exact extra-articular ligament being addressed in each case which limits the granularity of our analysis. Finally, limiting our analysis to 2-year follow-up may have prevented us from detecting other notable trends in complication rates. Regardless, this study offers a robust, population-based analysis of outcomes following concomitant ACL and EAL reconstruction.

## Conclusion

This analysis found low rates of serious medical complications following concomitant ACL and EAL reconstruction. Our cohort demonstrated greater rates of implant/graft failure, ankylosis, and lysis of adhesions than described following independent reconstruction of both structures. Concomitant reconstruction may be preferable to staged reconstruction as the latter method leads to insignificantly greater rates of implant/graft failure, osteoarthritis, and instability at various points of follow-up. However, despite slightly greater rates of orthopaedic complications following staged ACL and EAL reconstruction, both procedures are largely safe.

## Data Availability

No datasets were generated or analysed during the current study.
